# Supplementary material for: Innovations, contestations and fragilities of the health system response to COVID-19 in the Gauteng Province of South Africa
Source: PLoS One. 2021 Dec 17;16(12):e0261339. doi: 10.1371/journal.pone.0261339 (PMC8682886; doi:10.1371/journal.pone.0261339)
Supplement: S1 Appendix — (PDF) [file pone.0261339.s001.pdf]

INTERVIEW SCHEDULES

GAUTENG CITY REGION CASE STUDY ON THE HEALTH AND HEALTH SYSTEM RESPONSE TO COVID-19

Executive Managers

**Key informant code**	
**Date of interview**	
**Reminder that we may need to ask follow-up questions at a later stage**	

SECTION A: INTRODUCTION

1. Could you tell me very briefly about your role in the COVID-19 strategy and/or response in the Gauteng City Region (i.e. the Province of Gauteng)?

SECTION B: THE COVID-19 PANDEMIC RESPONSE IN THE GCR

2. Could you share with us the health and health system response to the COVID-19 pandemic in the Gauteng City Region?
    a. Is there a written document on the health and health system response? Could we have a copy of it?
    b. What are the key aspects/ elements of the response?
    c. Could you highlight the specific measures in the response that are/were envisaged to:
        i. Prevent the spread of COVID-19?
        ii. Limit or contain the spread of COVID-19?
        iii. Prepare the health system for a potential increase in COVID-19 infections and adapt actions as needed?
    d. Are there specific strategies in the COVID-19 response that focus on the health workforce? (probe planning, training and capacity building, mental health/ psychological support)
    e. Was there a dedicated budget allocated to the COVID-19 response or strategy?
    f. Have there been revisions/ amendment of the overall COVID-19 response/ strategy since the first version?

SECTION C: STAKEHOLDER INVOLVEMENT AND COMMUNICATION

3. Who were the main stakeholders involved in the development of the health and health system response to the COVID-19 pandemic? Probe:
    a. Other government departments/ entities
    b. Health facility managers
    c. Front-line health workers
    d. Organised labour

e. Civil society/ non-governmental organisations
f. Private sector
g. Other (please specify)

4. How did you communicate the GCR response to the various stakeholders?

SECTION D: PERSPECTIVES ON COVID-19 RESPONSE/ STRATEGY

5. What would you say are the main strengths of the COVID-19 response of the GCR? What do you think are the key factors that have contributed to these? (probe effectiveness, impact)
6. What are the weaknesses or failures in the COVID-19 response of the GCR? What contributed to these weaknesses?
7. Have there been any unintended consequences, either bad or good, because of the implementation of the original COVID-19 strategy? (Probe health equity, health service access, quality of care, crowding out of other services, etc.).
8. Were there any factors or issues that predisposed the GCR to the rapid increases in infections that were are experiencing now?
9. Could you comment on the political/ management interface (probe role of public servant, experts or scientific advisors)?

SECTION E: LESSONS FOR THE FUTURE

10. If you could turn back the clock, what would you have done differently in terms of the COVID-19 response?
11. What would you say are the key lessons from the management of COVID-19 or the implementation of the COVID-19 interventions? What are your recommendations regarding the future management of health crises (probe health system capacity, investment, sustainability, etc.)?
12. Are there any other comments that you wish to make?

THANK YOU VERY MUCH

Key Informants[1]

Key informant code	
**Date of interview**	

SECTION A: INTRODUCTION

1. Could you tell me very whether and how you have been involved in the COVID-19 strategy and/or response in the Gauteng City Region (i.e. the Province of Gauteng)?

SECTION B: PERSPECTIVES ON COVID-19 RESPONSE/ STRATEGY

2. What are your perspectives on the manner in which the COVID-19 pandemic has been managed in Gauteng
    a. What are the main strengths of the COVID-19 response of the GCR? What do you think are the key factors that have contributed to these?
    b. What are the weaknesses or failures in the COVID-19 response of the GCR? What contributed to these weaknesses?
    c. Have there been any unintended consequences (either positive or negative) of the way COVID-19 has been managed in Gauteng, (Probe health equity, health service access, quality of care, crowding out of other services, etc.).
3. Could you comment on the political/ management interface (probe role of public servant, experts or scientific advisors)?
4. If you were in charge of the health department, what would you have done to steer the management of COVID-19?

SECTION C: LESSONS FOR THE FUTURE

5. What would you say are the key lessons from the management of COVID-19 or the implementation of the COVID-19 interventions in the Province? What are your recommendations regarding the future management of health crises (probe health system capacity, investment, sustainability, etc.)?
6. Are there any other comments that you wish to make?

THANK YOU VERY MUCH

[1] Interview with Treasury-probe additional budget allocations; infrastructure development: Probe prioritisation of outstanding critical maintenance
