# Supplementary material for: Innovations, contestations and fragilities of the health system response to COVID-19 in the Gauteng Province of South Africa
Source: PLoS One. 2021 Dec 17;16(12):e0261339. doi: 10.1371/journal.pone.0261339 (PMC8682886; doi:10.1371/journal.pone.0261339)
Supplement: Attachment — Submitted filename: response to reviewer.pdf [file pone.0261339.s005.pdf]

UNIVERSITY OF THE WITWATERSRAND, JOHANNESBURG
WITS School of Public Health
Faculty of Health Sciences, 27 St Andrew's Road, Parktown 2193; Johannesburg; South Africa T +27 (0) 11 717 2043; www.wits.ac.za/hrhsarchi

28 October 2021

Nora Engel

Academic Editor: PLOS One

Dear Nora Engel

RE: RESPONSE TO REVIEWERS-PONE-D-21-14402

Title: Innovations, contestations and fragilities of the health system response to COVID-19 in the Gauteng Province of South Africa

We thank you and the reviewers for the comments on the above-mentioned manuscript that we received five months after submission. On behalf of my co-authors, this letter details the responses to the comments from the editor and the reviewers. The changes are highlighted in the text.

REVIEWER COMMENT	AUTHORS' RESPONSE	PAGE OR LINE REFERENCE
**GENERAL COMMENTS**		
Please ensure that your manuscript meets PLOS ONE's style requirements, including those for file naming.	We have checked and edited the paper, and ensured that the article is aligned with PLOS One's style requirements.<br>We have applied the PLOS One reference output style in the ***Endnote Programme*** throughout the paper.	**N/A**
When reporting the results of qualitative research, we suggest consulting the COREQ guidelines or other relevant checklists listed by the Equator Network, such as the SRQR, to	We have complied with these deadlines.	N/A

REVIEWER COMMENT	AUTHORS' RESPONSE	PAGE OR LINE REFERENCE
ensure complete reporting (http://journals.plos.org/plosone/s/submission-guidelines#locqualitative-research).		
In this case, please consider including more information on the number of interviewers, their training and characteristics.	We have included a separate heading entitled: *Research team and preparation for data collection*, as part of the section on Materials and Methods. We have described our training and experience.	Pages 16-17;<br>Lines 326-339
Moreover, please provide the interview guide used as a Supplementary file.	We have attached the two interview guides as supplementary material	
**REVIEWER 1-GENERAL COMMENTS TO EDITOR**		
The interview data was not provided given confidentiality concerns. Sound ethical processes were used to obtain consent etc.	This is a general comment in which the reviewer acknowledges that the research team adhered to sound ethical research conduct.	N/A
The paper portends to speak about the health sector but in effect speaks to the public health sector only; This is a challenge given that a large private health sector exists in the province and a significant portion of the population uses this sector.	We have addressed the reviewer's comment in the revised paper in several sections:<br>Introduction: overview of the two-tiered health system, and the private health sector in Gauteng (page 7, lines 133-139)<br>Study setting with overview of Covid-19 in Gauteng (page 12, lines 237-261).<br>Results: we have incorporated the views of the private sector respondents more prominently in themes (pages 34, 35, 38, 45 and 47), and also supported some of the views with evidence from the document analysis.<br><br>Although we interviewed three key informants from the private health sector, roughly the same number of people that we interviewed from the different stakeholder groups, we have included this as a study limitation.	Page 7, lines 133-139<br>Page 12, lines 237-261<br>Page 34, lines 613-622<br>Page 35, lines 642-644<br>Page 38, lines 707-709<br>Page 45, lines 868-877<br>Page 47, lines 916-921<br>Page 53, lines 1039-1040
As is usually the case key informants dont always have same views and opinions (largely depends on where they are positioned in the system). As a consequence there were many	Whose reality is the reviewer referring to?	Page 53, lines 1051-1053.

REVIEWER COMMENT	AUTHORS' RESPONSE	PAGE OR LINE REFERENCE
contradictory views, which leaves the reader uncertain to who reflects the reality! To assist the reader more quantitative data could have been used to support these views.	The value of the qualitative case study is that it highlights the innovations, the contestations and the fragilities in the COVID-19 response. We were deliberate in the choice of the word "contestations" as it reflects the opposing views and the issues of dispute among those interviewed. In the discussion, we have emphasised these contradictions and contestations in the key informant response and recommended further research	
**REVIEWER 1—IN-TEXT COMMENTS**		
**Abstract**. P.2 ln 23: also most populous province? this will also put the 30.5% into perspective for readers	We have revised the abstract to include this information	Page 2, Lines 23-25
**Introduction**		
P.4. ln 79: it should be noted however the relatively well performing health systems such as in the UK, Canada and Sweden were unable to contain the spread of COVID-19 or mitigate its impact	It is unclear what the relevance is of this reviewer comment.<br><br>In the original line 79, we highlight the importance of resilient and well-performing health systems in responding to the pandemic. It does not make any value judgement on the performance of the health systems of different countries.	N/A
P.5. ln 82: does the fact that it is a novel virus with rapid mutation not make this inevitable?	Again, it is unclear what the purpose of this comment is. The reviewer seems to suggest that the novel virus with rapid mutation is the cause of innovative and at times contradictory responses.<br><br>In the original line 82, we have summarised the 15 articles on country-level responses as "*complex, yet innovative and at times contradictory country-level responses*". These 15 articles are referenced clearly, hence any reader is able to read these articles, and may come to a different conclusion, or support our assessment.	N/A
P.5. ln 85: what about the social and cultural systems in a country? Have the countries in east not shown that these are as important as the health system?	The original lines 82-85 summarised the range of factors that influenced country-level responses, and we have referenced the editorial of a special issue of Health Policy and Technology that focused on the COVID-19 responses in more than 20 countries.	Page 4, lines 74-76.

REVIEWER COMMENT	AUTHORS' RESPONSE	PAGE OR LINE REFERENCE
	Nonetheless, we have rephrased the sentence as follows:<br>"The variations in country-level responses resulted in differential i*mpact of the pandemic during the first epidemic wave, further shaped by diverse political, social, cultural, economic and health system contexts"*	
P.6. ln 106: indeed - this suggests that exclusively focusing on the health system may not be sufficient.	This comment is unclear. Our paper does not suggest that the health system should be the only focus, BUT it remains a critical area of research focus and/or intervention.<br><br>Importantly, the combination of leadership, management and governance is a critical building block of the health system. We have presented it as the roof of our conceptual framework.	N/A
P.6. 116: what about South Korea, Taiwan Thailand? (THAILAND - WHO | World Health Organizationhttps://www.who.int › docs › country-case-studies)	We had included the South Korea in the literature review (see previous original reference 51).<br>Nevertheless, we have included a 2021 study on the experience of managing the pandemic in Thailand as follows:<br><br>"*A study on Thailand's response to COVID-19 found that the country's long-term investment in the health system resulted in an effective response that limited the spread of infections However, Thailand's pandemic response has been deficient in addressing the multiple social and economic dimensions of the crisis, engaging with various stakeholders and supporting community networks.*	Page 6, Lines 109-113.
P.7. ln 144: what about the roles of the metros - the City of Johannesburg and Tshwane for example also provide primary healthcare services	The original line 144 refers to the proportion of Covid-19 infections in the Gauteng Province. The reviewer's comment is unclear as we make no reference in that line to the provision of primary health care services.	Pages 12-13

REVIEWER COMMENT	AUTHORS' RESPONSE	PAGE OR LINE REFERENCE
	We have expanded on an overview of Gauteng under the heading "Study Setting".	
P.8. ln 151: would be useful to know if this refers to the public or private health sectors or both. the investment and dynamics in these sectors are very different and the private health sector in this province is large.	In this study, the health system response referred to the response by both public and private health sectors.<br><br>We have now clarified it in the introduction .We have included the proportion of the population with private health insurance in South Africa as a whole, as well as in the urban provinces of Gauteng and the Western Cape, as part of the introduction.	Pages 4-9
**Methods**: P.8. ln 160: maybe useful to explain what this means	We have rephrased the sentence as follows: *"The stated goals of the COVID-19 pandemic response*".	Page 9, line 182.
P.14. ln 255: in the SA context districts (especially health districts) are not synonymous with municipalities - with the latter being a sphere of government	We have now removed the word "municipalities" and left health districts in the text.	Page 15, line 299
Data analysis: P.19 ln 365: given the investment of the Office of the Premier in a favourable review was any pressure put on the researchers to make substantive (but not factual) changes to the report on which this case study is based?	The reviewer probably refers to line 356, rather than 365. The answer is "no".	N/A
**Results**		
P. 21 ln 390: given the size of the private health sector in Gauteng, why only 3 KIs were from this sector?	This was a qualitative case study, and we interviewed three senior or key policy actors in the private sector.	N/A
P. 22 ln 401: I found this table unnecessary - can the authors not summarise this data instead?	We strongly disagree with this comment. The table summarises the themes, sub-themes and links to the conceptual framework, and at a glance provides valuable information to the reader. We have revised the table to make it more readable.	Pages 24-26; Line 445
P. 29 ln 420: this may be the Commissioner	We have replaced "Commander" with "Commissioner	Page 27, Line 465.
P.30 ln 438: see note above - these are the same!	We have removed municipality, and left health district.	Page 27, line 458

REVIEWER COMMENT	AUTHORS’ RESPONSE	PAGE OR LINE REFERENCE
P.30 ln 444: any sense/data on the impact/value of this intervention? was it intersectoral or a health sector response?	Our case study focuses on the health system. In the results, we describe the views of key informants. It is standard practice that the writer or researcher makes no judgement on whether this was effective. This case study was not an impact study for which a different design and methods are required, apart from the fact that impact assessments can only be done after a period has elapsed.	N/A
P.30 ln 446: were these private sector KIs or public sector or a mixture?	We have inserted “public sector” before hospital managers	Page 28, Line 491
P.32 ln 488: would be useful to include data to support these views - for example on the rate of infections in healthcare workers	The previous line 488 refers to the central warehouse and dedicated budget for PPE. Nonetheless, we have inserted information on health workers infected under the relevant theme.	Page 44, lines 845-850.
P. 33 ln 505: these comments appear to contradict those in the section above - how can one make sense of this?	The theme is entitled Contestations and Contradictions, precisely to point out that there was no single narrative. The role of a good researcher is to present the views of participants as they are, including the contradictions and disagreements.	N/A
P. 34 ln 533: how does one reconcile these differing views?	See comment above. The aim of the paper was not to reconcile the views of the key informants.	N/A
P.40 ln 662: is this provincial health or LG or more widespread (other govt departments as well)?	Unclear comment. We have removed the sentence from the text.	N/A
P.40. ln 671: would be useful if the authors are able to quantify this impact on routine health services. The DHIS data can be used...	The purpose of the case study, as clearly outlined in the introduction and methods sections, was to describe the COVID-19 response in Gauteng and key informants perspectives. Our brief was not to quantify the impact on routine services. We have referenced papers that have done so in the discussion. See for example: *“A Gauteng study using routine health information found that both PHC and family planning utilisation declined significantly during the lockdown period”*	Page 51, lines 1001-1003.
P. 42 ln 701: which was?	We have inserted the Provincial Coronavirus Command Council, described earlier.	Page 41, line 768

REVIEWER COMMENT	AUTHORS’ RESPONSE	PAGE OR LINE REFERENCE
P. 44. Ln 764: again some data would be useful	We have inserted information released in August 2020 by the then Minister of Health on the COVID-19 infections among health workers, and referenced this information.	Page 44, lines 845-850.
**Discussion**		
P. 48 ln 846: challenge is that there are two health sectors in the province - but reported as one! Perhaps the authors should focus on the public health sector's response to the pandemic?	Our study included key informants from private sector. We asked all key informants about their involvement and their perspectives on the COVID-19 response in Gauteng. We have emphasised the missed opportunity of excluding the private health sector in the response.<br>We have revised the text extensively to include more information on the private health sector. See our response under general comments.	Page 7, lines 133-139<br>Page 12, lines 237-261<br>Page 34, lines 613-622<br>Page 35, lines 642-644<br>Page 38, lines 707-709<br>Page 45, lines 868-877<br>Page 47, lines 916-921<br>Page 53, lines 1039-1040
P.48 ln 857: yet the private sector KIs noted that they were excluded?	The comment is unclear<br>The original ln 856 -857 on p. 48 says<br>“…resource intensive Covid-19 response that marshalled the **entire public** health system to prevent infections, contain the pandemic and save lives.”	N/A
P.49 ln 858: apart from the premier's involvement - it is unclear how other government departments were involved in this response	We described the multi-sectoral response as part of Theme 1.	N/A
P. 49 ln 864: the funding had to come from somewhere - what has suffered as a consequence?	The important issue that we highlight is that the province injected additional financial resources into the response.	N/A
P. 49 ln 873: in addition, there were contradictions in the responses from KIs - how does one understand these?	Contestations is part of the title of the paper, it is a separate theme of the results, and we explain it in the discussion.	N/A
P. 51 ln 904: as noted above - this case study appears to be reflecting on the health system of the province (private and public) but in reality is only reflects on the public. This needs to be more clearly reflected throughout the paper - not only in the discussion section!	We disagree with the comment. We have revised the paper extensively to respond to this biased comment.	Page 7, lines 133-139<br>Page 12, lines 237-261<br>Page 34, lines 613-622<br>Page 35, lines 642-644<br>Page 38, lines 707-709<br>Page 45, lines 868-877<br>Page 47, lines 916-921<br>Page 53, lines 1039-1040

REVIEWER COMMENT	AUTHORS' RESPONSE	PAGE OR LINE REFERENCE
P.51 ln 919: Unit	Thank you for the comment. "unit" has been changed to Unit.	Page 51, line 1010
P.51 ln 923: It's curious that none of the KIs commented on the suspension/removal of the MEC, the impact of an acting MEC etc.?	The original line 923 refers to the discussion on unethical behaviours. We highlighted that many key informants mentioned that the corruption was a painful incident. The findings are direct quotes from the key informants, and we consider it inappropriate to mention names in the discussion.	N/A
P.54 ln 985: unclear why this is only mentioned towards the end of the document; what was the role/impact of the private health sector in the first wave? where its facilities better able to cope, etc?	It is incorrect to say that we only mention the private sector towards the end. We have highlighted that there were missed opportunities to engage with the private sector. In the discussion, we highlight the implications of the missed opportunity, and we highlight the stewardship role of government.<br><br>The revised paper contains more information on the private health sector in the introduction.	Page 7, lines 133-139<br>Page 12, lines 237-261<br>Page 34, lines 613-622<br>Page 35, lines 642-644<br>Page 38, lines 707-709<br>Page 45, lines 868-877<br>Page 47, lines 916-921<br>Page 53, lines 1039-1040
**REVIEWER 2**		
**General**		
This paper is well written, easy to follow and well-edited – a joy to read! The results are really interesting, and the value of the paper, as an in-depth description of a health system response to a shock is valuable. The recommendations are apt, and will be of value in the pandemic response moving forward.	We thank the reviewer for these positive comments.	N/A
The paper would be strengthened by either a clearly stated research question or a broader argument about what the authors wanted to learn from the exploration of the HS response. I definitely think there is value in a purely descriptive study, but also think that the reader's attention would be better directed by some indication of what specifically the authors wanted to learn.	We have revised the introduction, clarified the aim, study rationale and the research questions.	Pages 4-9

REVIEWER COMMENT	AUTHORS' RESPONSE	PAGE OR LINE REFERENCE
I feel that the sources of data could be better integrated – particularly the document analysis with the interview materials. Firstly, the document analysis is referred to explicitly in the discussion on Theme 3, but not Themes 1, 2, 4 or 5. Were there no contradictions evident in the documents, or innovations, for example?	We have noted the reviewer's comment, and have attempted to be explicit in the reference to the document analysis and the themes from the key informant interviews in the results section.	Pages 21-48
Secondly, the epidemiological data/ incidence rates does not seem to have informed the findings as presented in the results section. The authors state that this data was collected to "contextualise interview and provide a visual representation of the pandemic." However, I would have thought this data was available, and would not need to be collected or analysed by the authors as part of this study. I wonder why that data was included. Perhaps it would simplify the paper to position the epidemiological data as background/contextual information rather than as a core finding of the study, especially since the findings from the interviews and document analysis don't seem to be triangulated with or interpreted in light of this data.	We thank the reviewer for this comment. We have integrated the epidemiological information into the sub-section on "Study Setting".	Pages 12-13
Relatedly, a couple of times in the results section, it's not quite clear whether the finding is supported by the document analysis, interview data or epidemiological data (or all three). For example, ln 662-3, and 711-12	See comment above. We have removed the epidemiological trends as part of the methodology and results.	Pages 21-48
**Abstract**		
Pg 2, ln 38-39: The first line of the results section of the abstract feels more like a value judgement than a presentation of findings, and also feels at odds with the rest of the findings as presented in the abstract. I realise that this is one of the themes of the findings, but perhaps consider making that clear in the phrasing/structure of the paragraph: i.e. perhaps	We have rephrased the first sentence of the results section as follows:<br>*"Our case study found that Gauteng developed"*	Page 2, line 38.

REVIEWER COMMENT	AUTHORS' RESPONSE	PAGE OR LINE REFERENCE
rephrase to 'innovation and multi-sectoral collaboration was evident' or similar?		
**Introduction**		
Pg 4, ln 75 – Consider reporting excess deaths instead/in addition, to paint a clearer picture?	We have moved the original paragraph (lines 70-76). Given the contestations about excess deaths (the principal investigator at the South African Medical Research Council is the first person to highlight the limitations of modelling), we have updated the numbers of infections and deaths (as imperfect as these statistics might be), as these are reported to WHO, and daily in South Africa.<br><br>We have reported on rates and excess deaths in Gauteng in the section on "Study setting".	Pages 12-13
Pg 4 ln 70-76: Consider moving this paragraph down, so that the structure of the introduction goes from a broad, global-level discussion, and then introduces Africa, and South Africa more specifically. I.e. move to page 7, ln 127. It might also help to start the discussion of the South African case with a background paragraph that gives the reader the information they need about the political (e.g. quasi-federalism), economic and social context in South Africa. Could also include where the responsibility for health decisions lies. This information could also be included in 'study setting.'	We thank the reviewer for this suggestion.<br><br>We have implemented the suggestion of the reviewer, and moved the paragraph lower down. We have also provided additional information on South Africa, the health care system, and rephrased the entire section on South Africa.	Pages 7-9.
Pg 5, ln 90: I would include a 'at time of writing' caveat before this paragraph. Because, for example, Australia is currently seeing dramatic increases.	We have revised the introduction and ensured that that we refer to the response of a country <u>during the first wave</u>.	Page 5, lines 91-92.
Pg 7, ln 131: I suggest the reader needs more information here – i.e. 'resulted in corruption scandals including, for example….' The reference to '*the* reported corruption' suggests a) that there is only one, and that the reader already knows what it is.	We have rephrased the section, and updated information from studies that we accessed since the paper was submitted in April 2021.	Page 8, lines 149-160.

REVIEWER COMMENT	AUTHORS' RESPONSE	PAGE OR LINE REFERENCE
Pg 7, starting ln 136: This paragraph is doing 2 different things at one: firstly, it justifies the choice of Gauteng province as the case study, and then it justifies the study as whole (with reference to the shortage of empirical studies on this topic). I suggest, as above, that while the former justification is clear, the latter needs a paragraph of its own, in which the authors specify the phenomenon of interest more precisely.	In response to the reviewer, we have revised the introduction, and included the aim, study rationale, and the research questions.	Pages 4-9
Ln 132-134: Feels out of place in this paragraph – as above I suggest introducing a paragraph on background of SA for this type of information	We have revised the introduction in line with the reviewer's suggestion. We have included additional information on South Africa, and its health system.	
Ln 143: Include Gauteng proportion of population, so that the reader can interpret the weight of the percentage of cases.	We have expanded on the section on Study setting and included detailed information on Gauteng, and the epidemiology of COVID-19 in the province.	Pages 12-13
Pg 9, ln 184: This is a bit confusing because saving lives and prevention of deaths reads like an overall goal of the pandemic response, rather than one of the pillars. You description of it, starting at ln 193 makes sense, so, I wonder whether the issue is just the 'naming' of the pillar. Would something like 'service provision and treatment' make more sense?	We have renamed the pillar to "*health service preparedness and treatment*". We have included an additional sentence to cover the treatment part of the pillar.	Page 11, lines 214-218.
Pg 11, ln 229 – It's not quite clear why the 'framing' of policies was important to understand, and furthermore, the issue of framing isn't raised again in the paper.	We have rephrased the section as follows:<br>"*The purpose of the document analysis was twofold. Firstly, to examine the phrasing and content of COVID-19 policies and/or progress reports and the evolution of the pandemic response in Gauteng. Secondly, to allow for triangulation of various sources of data".*	Page 14, lines 271-275.
Pg 12, ln 229 – Because the phenomenon of interest for this study is not well defined, the reader wonders why it was important to understand the 'framing of the policy issues'	See comment above.	Page 14, lines 271-275.
Pg. 12, ln 230: Consider rephrasing to "to allow for triangulation of various sources of data". It doesn't make	We thank the reviewer for this suggestion, which we have implemented.	Page 14, Line 273

REVIEWER COMMENT	AUTHORS' RESPONSE	PAGE OR LINE REFERENCE
sense to say that you did document analysis to triangulate data from the document analysis.		
Pg 16, data analysis: It's not quite clear how the researchers assessed completeness prior to the analysis. In other words, without starting the analysis, how did the researchers know that data saturation had been reached? Is this perhaps more a point about data collection?	We rephrased this section as follows to avoid any misunderstanding. "*Following data collection, the principal researcher examined and collated all the raw data and submitted the collated data to the other researchers for review. The researchers held several meetings to discuss progress, debrief on the completed interviews, identify the emerging issues, identify gaps and assess whether new information emerged from the interviews.*	Page 18, lines 367-370
Pg 17, ln 325 – just for clarity was the consensus reached about the themes being appropriate and sufficient, i.e. that no new themes were emerging? Or that the researchers all agreed on the themes?	We have rephrased the paragraph as follows: "*Once the coding was complete, the four researchers held a meeting to discuss the independent codes and themes, and to reach inter-coder agreement. Following this, the research team held several other meetings to analyse the data, discuss and finalise the themes, their meaning and interpretation. We used an iterative process of inductive coding and theme development, followed by interrogation of these codes and themes in light of the study objectives and the selected conceptual framework*".	Page 19, lines 379-384.
Pg 18, ln 351: "and the documents analysed" – does this refer to triangulation between the interview data and the document review? The phrasing is a bit unclear.	The line refers to ensuring credibility. We have rephrased it as follows: "*The researchers ensured credibility by prolonged engagement with the documents, the key informant data and the audio recording of the interviews*"	Page 20, lines 409-410
**Results**		
Pg 39, ln 651: You write that "the central warehousing and procurement of PPE were a Gauteng innovation" – should this not then be referenced as a finding from the document analysis under theme 1?	This was reported by a key informant.	N/A
Discussion: g 48, ln 853 – "At face value" – I wonder whether the positioning here is that the Gauteng response was 'at face value' innovative, comprehensive and impressive, or whether it is more appropriate to the data to say that the	We thank the reviewer for this comment. We have rephrased it as follows: "*Both the document analysis and the key informant interviews revealed that the Gauteng province…..*"	Page 48, line 943

REVIEWER COMMENT	AUTHORS' RESPONSE	PAGE OR LINE REFERENCE
response was comprehensive and in some ways impressive, but that while some innovations were evident, there were also significant challenges and shortcomings. I feel that you don't want to imply that the innovations were illusory.		

Please do not hesitate to contact me should additional information be required.

Yours sincerely

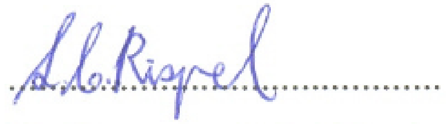

Professor Laetitia Rispel
Corresponding author
